# Highly Efficient *Agrobacterium*-Mediated Transformation of Tomato cv Micro-Tom From Cotyledon Explants

**DOI:** 10.21769/BioProtoc.5524

**Published:** 2025-12-05

**Authors:** Débora Pagliuso, Magdalena Rossi, Luciano Freschi

**Affiliations:** Botany Department, Institute of Biosciences, University of São Paulo, São Paulo, Brazil

**Keywords:** Plant transformation, Cotyledon, Tomato, *Agrobacterium*-mediated transformation, Stable transformation, Callus, Shoot regeneration

## Abstract

The tomato (*Solanum lycopersicum*) is a widely cultivated crop worldwide that serves as a model system for fruit development studies. *Agrobacterium tumefaciens*–mediated transformation of tomato has played a central role as a tool for analyzing the function of candidate genes and producing transgenic lines with enhanced resistance to pathogens, tolerance to abiotic stresses, and improved fruit quality traits. Among the many tomato varieties, the miniature dwarf cultivar Micro-Tom (MT) has been increasingly adopted as a model system for tomato research due to its short life cycle, small size, and high transformation efficiency. This protocol outlines a replicable methodology for *A. tumefaciens*–mediated transformation of Micro-Tom from cotyledon explants, utilizing cost-effective plant growth regulators for shoot regeneration, high transformation rates, reduced regeneration time, and enhanced rooting conditions.

Key features

• Highly efficient tomato genetic transformation from cotyledon explants (up to 80% efficiency rates).

• Fast and reproducible protocol optimized for the Micro-Tom cultivar covering acclimatization steps under greenhouse conditions.

• Utilizes cost-effective plant regulators for shoot regeneration.

## Background

Tomato (*Solanum lycopersicum*) is a fleshy fruit crop widely cultivated worldwide, providing a variety of health-promoting compounds for the human diet. Due to its small genome, amenability to stable genetic transformation, and the production of climacteric fleshy fruits, this species is a valuable model system for studies in plant physiology, genetics, and phytopathology, as well as multiple aspects of fruit physiology [1,2]. Tomato cultivars exhibit unique genetic backgrounds, which result in variations in their plant stature and architecture, as well as in fruit size and biochemical composition [3]. Among them, the miniature dwarf Micro-Tom (MT) cultivar offers the advantages of a small size (15–20 cm height), a short life cycle (~3 months), and the ability to grow in high densities [4,5]. The small size of this cultivar is primarily attributed to the *dwarf* and *self-pruning* mutations, which restrict brassinosteroid (BR) biosynthesis and result in a determinate growth habit, respectively [3].

Genetic transformation methods are classified as direct (biolistic, electroporation, and PEG-mediated) and indirect (*Agrobacterium sp*.), based on the mechanism of DNA delivery into plant cells. Among them, the most common methodology is the indirect protocol mediated by *Agrobacterium tumefaciens*, which utilizes the natural mechanism of this bacterium to transfer a backbone T-DNA into the plant genome. The transformation efficiency via this method is influenced by multiple factors, including the explant source and tissue age, the bacterial strain and concentration, the infection method and duration, and the plant hormones and chemicals used during co-culture and regeneration [6,7].

**Figure 1.**
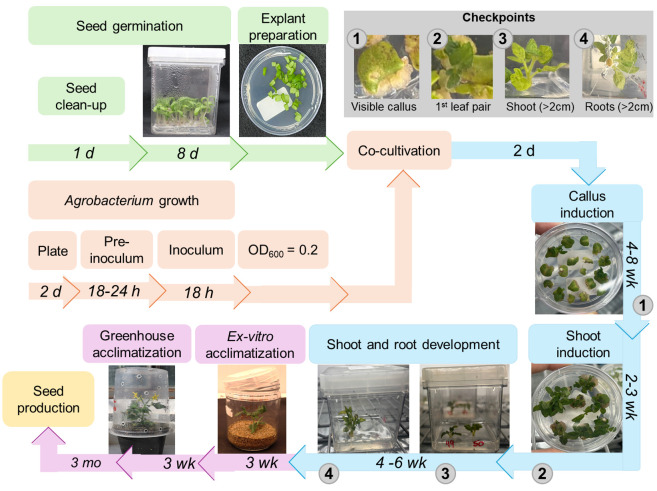
Flowchart for *Agrobacterium*-mediated transformation and regeneration of tomato (cv. Micro-Tom) using cotyledon explants. Bacterial inoculum preparation and seed germination steps are shown in orange and green, respectively. The shoot/root regeneration and the plant acclimatization steps are indicated in blue and purple, respectively. The estimated time required for each step is shown above the representative images. Gray circles indicate the requisites for transferring the explants to the next stage of the protocol. h: hours, d: days, mo: months, wk: weeks.

Tomato transformation is usually performed by using cotyledons of young seedlings as explants [8]. These cotyledons are co-cultivated with *Agrobacterium sp*. for the insertion of the transgene into the plant; then, by indirect organogenesis, new plants are regenerated and selected [9,10]. Efforts to optimize tomato transformation protocols have led to cumulative increases in transformation rates over the years [4,11,12]. This includes the use of various *A. tumefaciens* strains, such as LBA4404 [13,14], GH3101 [15], and EHA105 [16,17], with co-cultivation periods ranging from 1 to 3 days. The transformation efficiency reported for Micro-Tom generally depends on explant age, hormone regime, and bacterial strain [13,14,16]. Overall, the transformation rates of tomato typically range from 10% to 65%, depending on the type of explant and *Agrobacterium* strain [14,16–19]. Most protocols utilize the Murashige and Skoog medium [20] supplemented with zeatin (Z) and indole-3-acetic acid (IAA) at varying concentrations for the shoot regeneration step [21,22]. Additional chemicals, such as acetosyringone, have also been successfully tested to enhance efficiency and reduce the number of required steps [15–17,23–25]. As a critically important tool for both basic and applied research, tomato transformation protocols can significantly benefit from continuous technical refinements. Here, we describe a fast and reproducible *Agrobacterium*-mediated method optimized for the MT cultivar, with scalability for rapid analysis of gene function, with T0 plants produced in only 4–6 months (**Figure 1**). This method achieves high efficiency (up to 80%) using cotyledons as explants with the cost-effective replacement of zeatin (Z) for 6-benzylaminopurine (BA) throughout most of the shoot regeneration process. This transformation protocol has been employed to generate stable MT transgenic lines with constitutive and fruit-specific overexpression and RNAi-mediated silencing, as well as CRISPR-mediated knockout mutants, for multiple target genes, allowing the characterization of their functions on vegetative growth, flowering, and fruit development [28–36]. In all cases, homozygous transgenic lines were typically obtained within two or three generations, confirming transgene heritability. Combined with the rapid life cycle of the MT genotype, this relatively fast, straightforward, and highly efficient transformation method, which utilizes less expensive plant hormones, provides a robust system for functional genomics studies in tomato. It enables the analysis of gene function for a larger number of targets more quickly and at a lower cost than alternative protocols.

## Materials and reagents

**Biological materials**

1. *Agrobacterium tumefaciens* GV3101

2. *Solanum lycopersicum* cv Micro-Tom

**Reagents**

1. (NH_4_)NO_3_ (ACS Cientifica, CAS number: R10821000)

2. KNO_3_ (Sigma-Aldrich, CAS number: S31263-1KG)

3. MgSO_4_·7H_2_O (Synth, CAS number: S2035.01.AH)

4. KH_2_PO_4_ (Synth, CAS number: F2002.01.AG)

5. CaCl_2_·2H_2_O (Synth, CAS number: S1072.01.AH)

6. H_3_BO_3_ (Synth, CAS number: A1025.01.AH)

7. MnSO_4_·7H_2_O (Synth, CAS number: C2013.01.AH)

8. ZnSO_4_·7H_2_O (Synth, CAS number: S1072.01.AH)

9. KI (Sigma-Aldrich, CAS number: 30315-500G)

10. Na_2_MoO_4_·2H_2_O (Synth, CAS number: M1013.01.AE)

11. CuSO_4_·5H_2_O (Synth, CAS number: S105.01.AE)

12. CoCl_2_·6H_2_O (Synth, CAS number: C1044.01.AE)

13. FeSO_4_·7H_2_O (Synth, CAS number: S1057.01.AG)

14. Na_2_EDTA·2H_2_O (Sigma-Aldrich, CAS number: E5134-1KG)

15. Nicotinic acid (Synth, CAS number: A1043.01.AG)

16. Pyridoxine-HCl (Sigma-Aldrich, CAS number: P6280-10G)

17. Thiamine-HCl (Sigma-Aldrich, CAS number: T1270-25G)

18. Inositol (Sigma-Aldrich, CAS number: I5125-50G)

19. Sucrose (Synth, CAS number: S2609.01.AM)

20. Phytagel (Sigma-Aldrich, CAS number: P8169-1KG)

21. Agar (HIMEDIA, CAS number: RM301-500G)

22. Acetosyringone (Sigma-Aldrich, CAS number: D134406-5G)

23. 1-Naphthaleneacetic acid (Sigma-Aldrich, CAS number: M0640-25G)

24. Kanamycin (GIBCO, CAS number: 11815)

25. Zeatin (BioBasic, CAS number: 11815-032)

26. Meropenem (ABL Antibióticos do Brasil, CAS number: 1637-39-4)

27. 6-benzylaminopurine (Sigma-Aldrich, CAS number: B3408-5G)

28. Tryptone (HIMEDIA, CAS number: CR014-500G)

29. Yeast extract (KASVI, CAS number: RM301-500G)

30. NaCl (Synth, CAS number: C1060.01.AH)

31. Ethanol P.A (Synth, CAS number: A1083.07.BM)

32. Rifampicin (Sigma-Aldrich, CAS number: 13292-46-1)

33. Gentamycin (Sigma-Aldrich, CAS number: G3632-1G)

34. Polyoxyethylene sorbitan monolaurate (Tween 20) (Sigma-Aldrich, CAS number: P1379)

35. Commercial bleach (2%, v/v)

36. Distilled deionized water

37. HCl (37%) (Synth, CAS number: 13A0079.01.BJ)

38. NPK 10:10:10 (Heringer, CAS number: 66455-26-3)

39. Dolomite limestone (MgCO_3_ + CaCO_3_) (Calfertil, CAS number: 1317-65-3)

40. Peters 20:20:20 (Plantafol, Valagro, catalog number: F4)

41. Thermophosphate (Yoorin Master^®^, Yoorin Fertilizantes, catalog number: I-0009MG)

42. Substrate (Plantmax HT^®^, Eucatex)

43. Vermiculite (Nutriplan^®^, catalog number: 8000208)

**Solutions**

1. Macronutrients (Solution A) (see Recipes)

2. Calcium chloride (Solution B) (see Recipes)

3. Micronutrients (Solution C) (see Recipes)

4. FeEDTA (Solution D) (see Recipes)

5. Vitamins (see Recipes)

6. Acetosyringone (see Recipes)

7. Kanamycin (see Recipes)

8. Meropenem (see Recipes)

9. Naphthaleneacetic acid (NAA) (see Recipes)

10. 6-Benzylaminopurine (BA) (see Recipes)

11. Zeatin (see Recipes)

12. Peter’s solution 5× (see Recipes)

13. Plant media (see Recipes)

a. Germination medium (GM)

b. Virulence induction medium (VIM)

c. Shoot-inducing medium 1 (SIM-1)

d. Shoot-inducing medium 2 (SIM-2)

e. Shoot-inducing medium 3 (SIM-3)

f. Root-inducing medium (RIM)

g. Murashige and Skoog (MS) nutrient solution

h. Yeast extract peptone (YEP) medium

**Recipes**

**1. Macronutrients (Solution A)**

**Table:** 

Reagent	Final concentration	Quantity or volume
(NH_4_)NO_3_	33 g/L	33 g
KNO_3_	38 g/L	38 g
MgSO_4_·7H_2_O	7.4 g/L	7.4 g
KH_2_PO_4_	3.4 g/L	3.4 g
H_2_O		Up to 1,000 mL

Keep at 4 °C for up to two months.

**2. Calcium chloride (Solution B)**

**Table:** 

Reagent	Final concentration	Quantity or volume
CaCl_2_·2H_2_O	8.8 g/L	8.8 g
H_2_O		Up to 1,000 mL

Keep at 4 °C for up to two months.

**3. Micronutrients (Solution C)**

**Table:** 

Reagent	Final concentration	Quantity or volume
H_3_BO_3 _	1.24 g/L	1.24 g
MnSO_4_·7H_2_O	3.38 g/L	3.38 g
ZnSO_4_·7H_2_O	2.12 g/L	2.12 g
KI	0.166 g/L	0.166 g
Na_2_MoO_4_·2H_2_O	0.05 g/L	0.05 g
CuSO_4_·5H_2_O	0.005 g/L	0.005 g
CoCl_2_·6H_2_O	0.005 g/L	0.005 g
H_2_O		Up to 1,000 mL

Keep at 4 °C for up to two months.

**4. FeEDTA (Solution D)**

**Table:** 

Reagent	Final concentration	Quantity or volume
FeSO_4_·7H_2_O	2.78 g/L	2.78 g
Na_2_EDTA·2H_2_O	3.73 g/L	3.73 g
H_2_O		Up to 1,000 mL

Keep at 4 °C for up to two months.

**5. Vitamins**

**Table:** 

Reagent	Final concentration	Quantity or volume
Nicotinic acid	1 mg/mL	0.05 g
Pyridoxine-HCl	1 mg/mL	0.05 g
Thiamine-HCl	5 mg/mL	0.5 g
Inositol	50 mg/mL	5 g
H_2_O		Up to 50 mL

Keep at 4 °C for up to two months.

**6. Acetosyringone**

**Table:** 

Reagent	Final concentration	Quantity or volume
Acetosyringone	100 mM	196 mg
Ethanol		Up to 10 mL

Filter sterilize, aliquot, and store at -20 °C for up to three months.

**7. Kanamycin**

**Table:** 

Reagent	Final concentration	Quantity or volume
Kanamycin	50 mg/mL	1.25 g
H_2_O	-	Up to 25 mL

Filter-sterilize, aliquot, and store at -20 °C for up to three months.

**8. Meropenem**

**Table:** 

Reagent	Final concentration	Quantity or volume
Meropenem	8.3 mg/mL	1 g
H_2_O		Up to 120 mL

Filter-sterilize, aliquot, and store at -20 °C for up to three months. Be aware of the expiration date of the antibiotic.

**9. Naphthaleneacetic acid (NAA)**

**Table:** 

Reagent	Final concentration	Quantity or volume
NAA	0.4 mM	0.0074 g
H_2_O		Up to 100 mL

Add 1 M KOH drops to dissolve, then filter-sterilize or autoclave. Aliquot and store at 4 °C for up to six months.

**10. 6-Benzylaminopurine (BA)**

**Table:** 

Reagent	Final concentration	Quantity or volume
BA	0.5 mM	0.00225 g
H_2_O		Up to 200 mL

Add some 1 N HCl drops to dissolve, then filter-sterilize, aliquot, and store at -20 °C for up to one year.

**11. Zeatin**

**Table:** 

Reagent	Final concentration	Quantity or volume
Zeatin	1 mM	0.0219 g
H_2_O		Up to 100 mL

Add some 1 N HCl drops to dissolve, then filter-sterilize, aliquot, and store at -20 °C for up to six months.

**12. Peter’s solution 5×**

**Table:** 

Reagent	Final concentration	Quantity or volume
Peters 20:20:20 (N:P:K)	3.5 g/L	3.5 g
H_2_O		Up to 1,000 mL

Dilute in tap water and use within one week. Keep at 4 °C.

**13. Plant media**

**a. Germination medium (GM)**

**Table:** 

Reagent	Final concentration	Quantity or volume
Solution A	2.5% (v/v)	25 mL
Solution B	2.5% (v/v)	25 mL
Solution C	0.25% (v/v)	2.5 mL
Solution D	0.5% (v/v)	5 mL
Vitamin	0.1% (v/v)	1 mL
Sucrose	1.5% (w/v)	15 g
Phytagel	0.23% (w/v)	2.3 g
H_2_O		Up to 1,000 mL

Adjust pH to 5.8 with KOH and sterilize the media by autoclaving at 121 °C for 15 min and 1.5 atm. In a biosafety cabinet, add approximately 15 mL of the media to each sterilized 15 × 50 mm plate. Keep the media at 4 °C for up to two weeks.

**b. Virulence induction medium (VIM)**

**Table:** 

Reagent	Final concentration	Quantity or volume
Solution A	5% (v/v)	50 mL
Solution B	5% (v/v)	50 mL
Solution C	0.5% (v/v)	5 mL
Solution D	1% (v/v)	10 mL
Vitamin	0.1% (v/v)	1 mL
Sucrose	3% (w/v)	30g
Agar	0.6% (w/v)	6 g
Acetosyringone 100 mM	0.1 mM	1 mL
NAA 0.4 mM	0.0004 mM	1 mL
H_2_O		Up to 1,000 mL

Adjust pH to 5.8 with KOH and sterilize the media by autoclaving at 121 °C for 15 min and 1.5 atm. Then, after partial cooling, add the acetosyringone and NAA. In a biosafety cabinet, add approximately 15 mL of media to each sterilized 15 × 50 mm plate. Keep the media at 4 °C for up to two weeks.

**c. Shoot-inducing medium 1 (SIM-1)**

**Table:** 

Reagent	Final concentration	Quantity or volume
Solution A	5% (v/v)	50 mL
Solution B	5% (v/v)	50 mL
Solution C	0.5% (v/v)	5 mL
Solution D	1% (v/v)	10 mL
Vitamin	0.1% (v/v)	1 mL
Sucrose	3% (w/v)	30 g
Agar	0.6% (w/v)	6 g
Kanamycin 50 mg/mL	0.05 mg/L	1 mL
Zeatin 0.5 mM	0.01 mM	20 mL
Meropenem 8.3 mg/mL	0.03 mg/L	3.6 mL
H_2_O		Up to 1,000 mL

Adjust pH to 5.8 with KOH and sterilize the media by autoclaving at 121 °C for 15 min and 1.5 atm. Then, after partial cooling, add the hormones and antibiotics. In a biosafety cabinet, add approximately 15 mL of media to each sterilized 15 × 50 mm plate. Keep the media at 4 °C for up to two weeks.

**d. Shoot-inducing medium 2 (SIM-2)**

**Table:** 

Reagent	Final concentration	Quantity or volume
Solution A	5% (v/v)	50 mL
Solution B	5% (v/v)	50 mL
Solution C	0.5% (v/v)	5 mL
Solution D	1% (v/v)	10 mL
Vitamin	0.1% (v/v)	1 mL
Sucrose	3% (w/v)	30g
Agar	0.6% (w/v)	6 g
Kanamycin 50 mg/mL	0.1 mg/L	2.4 mL
Zeatin 0.5 mM	0.003 mM	6 mL
Meropenem 8.3 mg/mL	0.03 mg/L	3.6 mL
H_2_O		Up to 1,000 mL

Adjust pH to 5.8 with KOH and sterilize the media by autoclaving at 121 °C for 15 min and 1.5 atm. Then, after partial cooling, add the phytohormones and antibiotics. In a biosafety cabinet, add approximately 40 mL of media to each sterilized Magenta^TM^ vessel. Keep the media at 4 °C for up to two weeks.

**e. Shoot-inducing medium 3 (SIM-3)**

**Table:** 

Reagent	Final concentration	Quantity or volume
Solution A	5% (v/v)	50 mL
Solution B	5% (v/v)	50 mL
Solution C	0.5% (v/v)	5 mL
Solution D	1% (v/v)	10 mL
Vitamin	0.1% (v/v)	1 mL
Sucrose	3% (w/v)	30 g
Agar	0.6% (w/v)	6 g
Kanamycin 50 mg/mL	0.1 mg/L	2.4 mL
BA 0.5 mM	0.005 mM	10 mL
Meropenem 8.3 mg/mL	0.0075 mg/L	0.9 mL
H_2_O		Up to 1,000 mL

Adjust pH to 5.8 with KOH and sterilize the media by autoclaving at 121 °C for 15 min and 1.5 atm. Then, after partial cooling, add the hormones and antibiotics. In a biosafety cabinet, add approximately 40 mL of media to each sterilized Magenta^TM^ vessel. Keep the media at 4 °C for up to two weeks.

**f. Root-inducing medium (RIM)**

**Table:** 

Reagent	Final concentration	Quantity or volume
Solution A	2.5% (v/v)	25 mL
Solution B	2.5% (v/v)	25 mL
Solution C	0.25% (v/v)	2.5 mL
Solution D	0.5% (v/v)	5 mL
Vitamin	0.1% (v/v)	1 mL
Sucrose	3% (w/v)	30 g
Phytagel	0.23% (w/v)	2.3 g
Meropenem 8.3 mg/mL	0.0075 mg/L	0.9 mL
H_2_O		Up to 1,000 mL

Adjust the pH to 5.8 with KOH and sterilize the media by autoclaving at 121 °C for 15 min at 1.5 atm. Then, after partial cooling, add the hormones and antibiotics. In a biosafety cabinet, add approximately 40 mL of the media to each sterilized 15 × 100 mm plate. Keep the media at 4 °C for up to two weeks.

**g. Murashige and Skoog (MS) nutrient solution**

**Table:** 

Reagent	Final concentration	Quantity or volume
Solution A	2.5% (v/v)	25 mL
Solution B	2.5% (v/v)	25 mL
Solution C	0.25% (v/v)	2.5 mL
Solution D H_2_O	0.5% (v/v)	5 mL Up to 1,000 mL

Adjust the pH to 5.8 with KOH and keep at 4 °C for up to two months.

**h. Yeast extract peptone (YEP) medium**

**Table:** 

Reagent	Final concentration	Quantity or volume
Tryptone	10 g/L	10 g
Yeast extract	10 g/L	10 g
NaCl	5 g/L	5 g
Agar	9.6 g/L	15 g
H_2_O		Up to 1,000 mL

Adjust pH to 7.0 with NaOH and sterilize the media by autoclaving at 121 °C for 15 min and 1.5 atm. In a biosafety cabinet, add approximately 40 mL of the media to each sterilized 15 × 100 mm plate. Keep the media at 4 °C for up to two weeks.

**Laboratory supplies**

1. Graduated cylinder (100 mL) (Laborglass, catalog number: 9138624)

2. Becker (600 mL) (Laborglass, catalog number: 9110648)

3. Metallic sieve (mesh 100) (ASTM 100, Prolab, catalog number: BEIP022)

4. Metallic spatula (20 cm) (Prolab, catalog number: MER063-1)

5. Plastic petri dish (15 × 100 mm and 15 × 50 mm) (J.Prolab)

6. Fine pointed tweezers (Style AA) (Sigma-Aldrich, catalog number: Z680184)

7. Scissors (Sigma-Aldrich, catalog number: Z265977)

8. Falcon (50 mL) (Corning, catalog number: 430829)

9. Micropipettes (Eppendorf) and tips (Axygen)

10. Magenta^TM^ vessel (Merck, catalog number: V8505, dimension 77 mm × 77 mm × 97 mm)

11. Jars with screw cap for tissue culture (Bio-Sama, catalog number: PP85)

## Equipment

1. Centrifuge (ThermoScientific, model: Sorvall ST16R, rotor 75003658–21 cm, 50 mL tube capacity)

2. Bacterial incubator and shaker (Marconi, model: MA832/1)

3. Autoclave (Av plus, Phoenix Luferco)

4. Spectrophotometer (UV 5100 UV/Vis, METASH, Shanghai Inc.)

5. Biosafety cabinets (Esco class II BSC streamline and AMD solutions)

6. Orbital shaking table (TS 2000A VDRL shaker, biomixer)

7. Plant growth chamber (Environplant, Instalafrio)

## Procedure

**A. Seed preparation and germination**

*Note: Procedures must be performed in a biosafety cabinet. For each transformation batch, it is recommended to use 20 plates, each plate with 15 explants, for a total of 300 explants, which require approximately 100 seedlings. Usually, one trained person can manage to perform up to three transformation batches per week simultaneously.*

**Figure 2.**
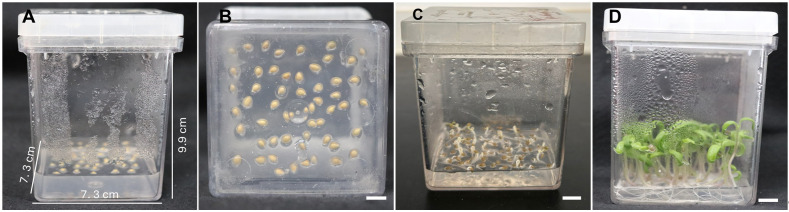
In vitro seed germination. (A, B) Magenta vessel with the inoculated seeds. (C) Four-day-old etiolated seedlings before transferring to light conditions. (D) Seedlings used as a source of explants for transformation. Scale bar = 1 cm.

1. All the glasses, micropipettes, tips, media, sieves, and distilled water must be sterilized by autoclaving. The spatula and tweezers must be heat-sterilized before use; this can be done by autoclaving or using a microbead sterilizer with glass beads at 250 °C for 5 min.

2. In a 250 mL beaker, add 80 mL of sterile distilled water, 20 mL of commercial bleach, and three drops of Tween 20, then homogenize.

3. Place an orbital shaking table inside the biosafety cabinet, add the seeds to the solution, and slowly agitate (~60 rpm) for ~15 min to promote uniform disinfestation without damaging the seed coat. Excess agitation can reduce germination rates.

4. Using a sterile metallic sieve, discard the solution and retain the seeds. Wash the seeds with sterile distilled water to remove the residual bleach and Tween 20. For this, add approximately 100 mL of sterile distilled water to the beaker containing the seeds and agitate on an orbital shaker (~60 rpm) for ~5 min. Recover the seeds with a sterile sieve and wash them again nine more times as described.

5. At the last wash, leave it to rest for ~30 min in the water.

6. Remove the seeds using a sterile sieve and discard the excess water.

7. Inoculate ~40 seeds into a Magenta vessel containing GM (see Recipes) (Figure 2A, B). Place the seeds at a distance of ~0.5 cm from each other.

8. Keep the seeds in the dark at 25 °C for 4 days (Figure 2C).

9. Transfer them to light conditions (12-h photoperiod, ~50 μmol·m^-2^·s^-1^, 25 ± 1 °C) for an additional 4-day period (Figure 2D).

**B. *Agrobacterium tumefaciens* cultivation and preparation**

*Note: Procedures must be performed in a biosafety cabinet.*

1. Streak the *Agrobacterium tumefaciens* strain carrying the construction of interest in a plate containing solid YEP medium supplemented with appropriate antibiotics (see Recipes) and incubate at 28 ± 1 °C for 2 days to get isolated colonies.

2. Prepare a pre-inoculum by inoculating 3 mL of YEP media supplemented with appropriate antibiotics with one bacterial colony. Incubate at 28 ± 1 °C and approximately 180 rpm for 18–24 h in the dark.

3. Prepare the inoculum by adding 100–300 μL of the pre-inoculum to 250-mL Erlenmeyer flasks containing 50 mL of YEP medium supplemented with the appropriate antibiotics and incubating overnight under the same conditions. The remaining pre-inoculum should be inactivated by autoclaving.

4. Once the OD_600_ reaches 0.5–0.7, centrifuge the culture into a 50-mL plastic tube at 3,000× *g* for 10 min at room temperature (25 ± 1 °C). Resuspend the pellet with liquid GM to an OD_600_ of 0.2–0.3. Store the suspension in the dark and at room temperature while the explants are prepared.

**Critical step:** The bacterial density at this stage is essential for successful transformation. Overgrown cultures (OD_600_ > 0.8) may cause tissue necrosis and decrease transformation efficiency. Adjust culture density carefully before co-incubation.

**C. Explant and co-incubation**

*Note: Procedures must be performed in a biosafety cabinet.*

**Figure 3.**
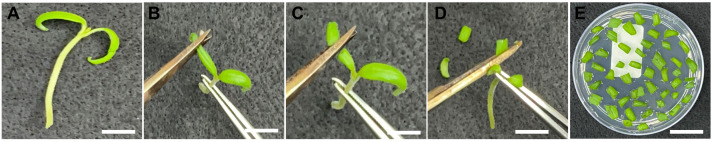
Cotyledon explant isolation. (A) Seedling used for cotyledon removal. (B, C) Removal of the extremities of each cotyledon and subsequent transversal cut to generate two cotyledon pieces. (D) Explant isolation. (E) Explants used for co-incubation with *Agrobacterium tumefaciens*. Scale bar = 1 cm.

1. Using sterilized tweezers and scissors, hold the cotyledon and cut both extremities, subsequently making a transversal cut to generate two cotyledon pieces (Figure 3). Add 40 explants with the abaxial portion of the cotyledons facing down to each plate containing VIM (see Recipes). Explants can be cut directly onto the VIM plates and then rearranged to maintain a distance of approximately 2 mm between them.

2. In each plate, add approximately 4 mL of the *A. tumefaciens* suspension with a sterile pipette. Incubate for 30 min in the dark without agitation at room temperature, then remove the suspension using new tips and a pipette. Seal the plates and maintain them at 25 ± 1 °C in the dark for 2 days.

**Caution**: Avoid excess bacterial suspension during co-cultivation, as residual bacterial suspension can promote excessive bacterial growth and tissue necrosis.

**Critical step**: Maintain explants with the abaxial side facing down during infection and regeneration. Incorrect orientation drastically reduces shoot induction rates.

**D. Plant regeneration**

*Note: Procedures must be performed in a biosafety cabinet. All containers must be autoclaved before use. Tissue culture for plant regeneration should be maintained under controlled conditions: 12-h photoperiod, ~50 μmol·m^-2^·s^-1^, and 25 ± 1 °C.*

**Figure 4.**
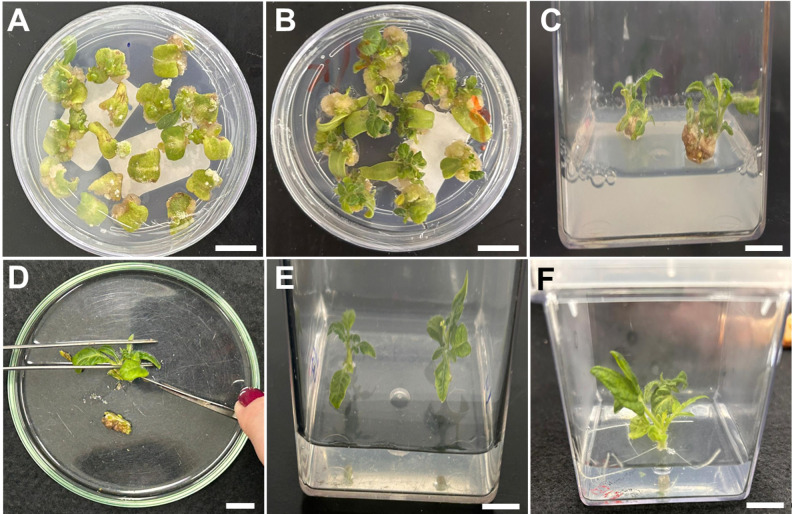
Shoot and root regeneration steps. (A) Callus formation (SIM-1). (B) Callus formation and initial shoot formation (SIM-1). (C) Shoot development (SIM-2). (D) Removal of callus. (E) Shoot expansion after callus removal (SIM-3). (F) Root induction (RIM). Scale bars = 1 cm. SIM, shoot-inducing medium; RIM, root-inducing medium.

**Figure 5.**
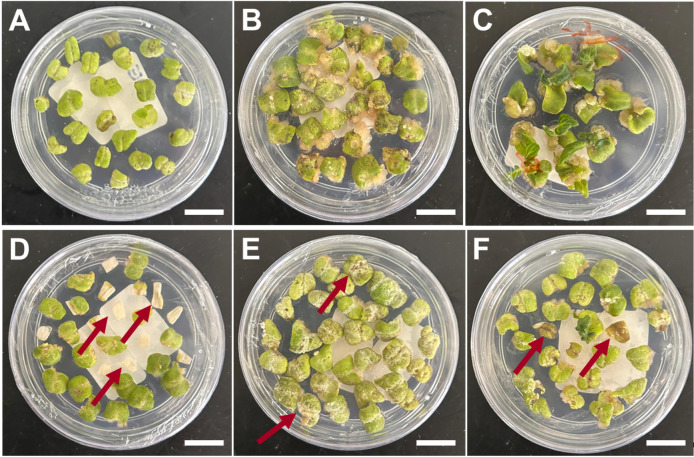
Identification of viable and unviable explants at callus formation and initial shoot formation stages. (A) Cotyledon explant after co-incubation. (B) Explants producing visible callus. (C) Initial shoot development. (D) Dead explant before cellular differentiation. (E) Brownish and white spots reveal unviable explants at callus formation. (F) Progressive death of the untransformed explants at the shoot formation stage. A–C, healthy explants. D–F, damaged and unviable explants. Read arrows indicate damaged, not viable tissues. Scale bars = 1 cm.

1. After the 2-day period of co-incubation, transfer the explants to plates containing SIM-1 (see Recipes), ensuring the abaxial portion of the cotyledon remains in contact with the medium. Keep the explants for 2 weeks at 25 ± 1 °C in the light for shoot development. After two weeks, transfer the explants to a new SIM-1-containing plate and incubate for an additional two weeks (Figure 4A, B). Healthy explants typically exhibit a compact, green-brownish callus with visible meristematic domes after 4 weeks on SIM-1 medium (Figure 5A–C). Non-viable explants become necrotic (Figure 5D–F). Under antibiotic selection, resistant tissue remains green and proliferative, whereas non-resistant tissue bleaches and undergoes progressive necrosis.

2. Transfer the regenerating explants to Magenta vessels containing SIM-2 (see Recipes) (Figure 4C).

3. After 2–3 weeks, when the shoots reach 1–2 cm, transfer the explants to Magenta vessels containing SIM-3 (see Recipes).

4. As soon as the regenerated shoots exhibit 1–3 fully developed leaves, isolate the shoot tissues from the callus using a sterile scalpel under aseptic conditions (Figure 4D). Discard the callus tissues and transfer the isolated shoots to Magenta vessels containing RIM (see Recipes) (Figure 4E).

5. Every two weeks, transfer the regenerated shoots to Magenta vessels containing fresh RIM media to ensure an adequate nutrient supply to promote root development. The transfer of the regenerated plants to ex vitro acclimation should be initiated as soon as one or more roots (>2 cm long) are formed (Figure 4F).

**Critical step:** During the transfers between regeneration media, carefully remove necrotic tissues. Cutting the base at a 45° angle improves rooting and uniform growth.

**E. Ex vitro hardening of the regenerated plants**

**Figure 6.**
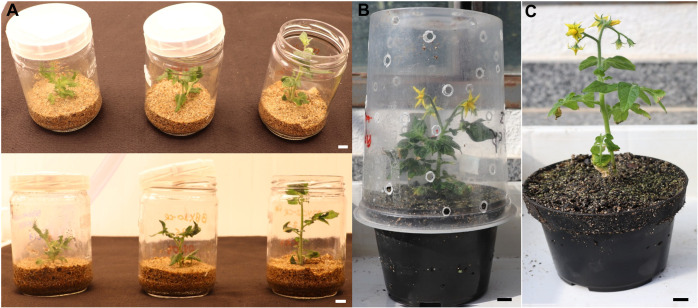
Ex vitro acclimatization of the regenerated plants. (A) Gradual exposure of the regenerated plants to ex vitro conditions. (B, C). Plant growth under standard greenhouse conditions. Scale bars = 1 cm.

1. Carefully remove the rooted plants from the Magenta vessels and wash them in distilled water to remove any medium residues.

2. Transfer one regenerated plant to each jar containing vermiculite saturated with MS nutrient solution (see Recipes) and completely cover the jar with a lid (Figure 6A). Keep the jars under mild growth conditions (12-h photoperiod, ~100 μmol·m^-2^·s^-1^, 25 ± 1 °C) for 3 weeks, routinely watering with MS nutrient solution to maintain the vermiculite moist. In the first week, keep the jar fully closed. In the second week, open half of the lid, and in the third week, completely remove the lid to promote the progressive hardening of the plants (Figure 6A).

3. Transfer the plants to 250-mL pots with a 1:1 mixture of commercial substrate and expanded vermiculite, supplemented with 1 g/L of NPK 10:10:10, 4 g/L of dolomite limestone (MgCO_3_ + CaCO_3_), and 2 g/L thermophosphate, and keep under greenhouse conditions for final acclimatization. Initially, maintain the pots protected from excessive dehydration and direct light by covering them with plastic cups with small holes and 50% shade cloth (Figure 6B). After one week, remove the plastic cups and the shade cloth, and maintain the plants under standard greenhouse conditions for seed production (Figure 6C).

**Caution**: Gradual lid removal during acclimatization is essential to prevent sudden plant desiccation.

**Critical step**: Ensure the complete removal of culture medium from the roots before transferring to the substrate to avoid fungal growth.

## General notes and troubleshooting

**Troubleshooting**

**A. Seed preparation and germination**

1. In case of frequent fungal/bacterial contamination during the seed germination step, increase 2–3 times the concentration of commercial bleach at the seed disinfestation step. The complete removal of the pulp during seed cleaning is also key to minimizing fungal/bacterial contamination during in vitro tomato seed germination.

2. In case of low seed germination, the concentration of commercial bleach can be reduced by half.

3. The incubation under absolute darkness can be prolonged by up to three days if the seed germination rates are lower than 50%.

**B. *Agrobacterium tumefaciens* cultivation and preparation**

1. If the bacterial culture is not growing, evaluate the transgene stability in the bacterial stock by PCR. If the problem persists, verify the antibiotic concentrations in use. The antibiotic aliquots should not be exposed to the UV light of the biosafety chamber or refrozen. During solid media preparation, antibiotics should be added when the media reaches approximately 45 °C to avoid thermal degradation.

2. In case of excessive necrosis or death of the explants soon after co-incubation, the optical density of the bacterial solution can be reduced by half.

**C. Explant and co-incubation**

1. In case of low transformation efficiency, other *A. tumefaciens* strains can be used, such as EHA105 and EHA101.

**D. Plant regeneration**

1. In case of persistent *A. tumefaciens* growth during shoot regeneration, wash the explants with a solution of sterile MS solution containing 0.03 mg/L (v/v) meropenem.

2. In case of low regeneration, the kanamycin concentration can be reduced by half; however, this change may increase the number of false positives. Also, verify the validity of the antibiotic and hormone stock solution (stable for 6 months at -20 °C). Do not refreeze the aliquots of the antibiotic and hormone stock solution.

3. In case of low regeneration, the optical concentration of *A. tumefaciens* can be increased to 0.6–1.0.

4. In case of the same explant generating two or more buds, select one to follow the next steps and discard the other one. This is performed to ensure independent transformation events are obtained.

**E. Ex vitro hardening of the regenerated plants**

1. In case of high rates of false positives (higher than 20%), increase the kanamycin concentration by 50% during the shoot regeneration steps.

2. In case of fungal attack during plant acclimatization, remove the contaminated tissues with scissors or a scalpel and apply appropriate fungicides.

## Validation of protocol

**Figure 7.**
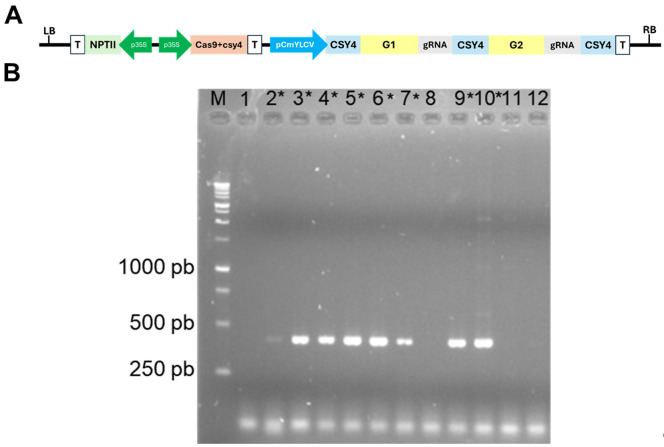
Validation of the tomato (cv. Micro-Tom) transformation. (A) Vector designed to create defined deletions for the target gene-coding sequences using two sgRNAs alongside the Cas9 endonuclease gene, based on the pDIRECT22 vector. LB: left border; RB: right border; T: terminator; *p35S*: cauliflower mosaic virus *35S* promoter; *pCmYLCV*: Cestrum yellow leaf curling virus promoter. G1 and G2: guide RNA-specific sequences; csy4: specific single-turnover RNA endoribonuclease derived from type I-F CRISPR systems of *Pseudomonas aeruginosa; NPTII*: NEOMYCIN PHOSPHOTRANSFERASE II (kanamycin-resistance gene). (B) PCR analysis for the detection of the presence of the transgene. The first lane is the DNA ladder. The expected product size is 440 pb. The numbers 1–12 represent transformant samples, and the positive samples are marked with an asterisk (*).

This protocol was used to generate numerous transgenic lines, as reported in Moreira et al. (2025) [26], Shiose et al. (2024) [27], Zuccarelli et al. (2023) [29], Lira et al. (2023) [30], Lira et al. (2022) [32], Alves et al. (2020) [33], Rosado et al. (2019) [34], Lupi et al. (2019) [35], and Bianchetti et al. (2018) [36]. Transgene heritability has been confirmed in all cases analyzed. To provide further validation of the protocol, Micro-Tom transformation was performed using a vector designed to create defined deletions for the target gene-coding sequences using two sgRNAs alongside the Cas9 endonuclease gene—vector pDIRECT22C (Figure 7A)—as described in Cermak et al. (2017) [37].

PCR analysis was conducted to confirm the presence of the transgene in the regenerants (Figure 7B). For this, young leaves of the regenerated plants were submitted to genomic DNA (gDNA) extraction following the Edwards’ protocol [38]. The extracted gDNA was submitted to PCR with Taq DNA polymerase (Invitrogen^®^) following manufacturers recommendations (Buffer 1×, 0.2 mM each dNTP, 1.5 mM MgCl_2_, 0.5 μM primer forward and reverse, taq DNA polymerase 1 U, DNA template 30 ng) under thermal cycler conditions of initial denaturation of 94 °C for 5 min, 35 cycles of 94 °C for 30 s, 55 °C for 20 s, 72 °C for 30 s, and final extension of 72 °C for 5 min. The oligonucleotides used to amplify a segment of the Cas9 endonuclease-encoding gene were 5′-CTCTGATGTGGATAAGTTGTTC-3′ and 5′-CTGAGAGTGGAGCCTTGGTG -3′. The PCR products were analyzed by electrophoresis with 1% agarose gel at 90 V. In the example illustrated in Figure 7B, out of the 12 samples evaluated, eight were confirmed as positive, thus carrying the Cas9 endonuclease-encoding gene with a product size of 440 bp.
